# L-glutamate released from activated microglia downregulates astrocytic L-glutamate transporter expression in neuroinflammation: the ‘collusion’ hypothesis for increased extracellular L-glutamate concentration in neuroinflammation

**DOI:** 10.1186/1742-2094-9-275

**Published:** 2012-12-23

**Authors:** Junpei Takaki, Koki Fujimori, Marie Miura, Takeshi Suzuki, Yuko Sekino, Kaoru Sato

**Affiliations:** 1 Laboratory of Neuropharmacology, Division of Pharmacology, National Institute of Health Sciences, 1-18-1 Kamiyoga, Setagaya-ku, Tokyo 158-8501, Japan; 2 Division of Basic Biological Science, Faculty of Pharmacy, Keio University, 1-5-30 Shiba-koen, Minato-ku, Tokyo 105-8512, Japan

**Keywords:** L-glutamate, Microglia, Transporter, Astrocytes, Inflammation, Hemichannel

## Abstract

**Background:**

In the central nervous system, astrocytic L-glutamate (L-Glu) transporters maintain extracellular L-Glu below neurotoxic levels, but their function is impaired with neuroinflammation. Microglia become activated with inflammation; however, the correlation between activated microglia and the impairment of L-Glu transporters is unknown.

**Methods:**

We used a mixed culture composed of astrocytes, microglia, and neurons. To quantify L-Glu transporter function, we measured the extracellular L-Glu that remained 30 min after an application of L-Glu to the medium (the starting concentration was 100 μM). We determined the optimal conditions of lipopolysaccharide (LPS) treatment to establish an inflammation model without cell death. We examined the predominant subtypes of L-Glu transporters and the changes in the expression levels of these transporters in this inflammation model. We then investigated the role of activated microglia in the changes in L-Glu transporter expression and the underlying mechanisms in this inflammation model.

**Results:**

Because LPS (10 ng/mL, 72 h) caused a significant increase in the levels of L-Glu remaining but did not affect cell viability, we adopted this condition for our inflammation model without cell death. GLAST was the predominant L-Glu transporter subtype, and its expression decreased in this inflammation model. As a result of their release of L-Glu, activated microglia were shown to be essential for the significant decrease in L-Glu uptake. The serial application of L-Glu caused a significant decrease in L-Glu uptake and GLAST expression in the astrocyte culture. The hemichannel inhibitor carbenoxolone (CBX) inhibited L-Glu release from activated microglia and ameliorated the decrease in GLAST expression in the inflammation model. In addition, the elevation of the astrocytic intracellular L-Glu itself caused the downregulation of GLAST.

**Conclusions:**

Our findings suggest that activated microglia trigger the elevation of extracellular L-Glu through their own release of L-Glu, and astrocyte L-Glu transporters are downregulated as a result of the elevation of astrocytic intracellular L-Glu levels, causing a further increase of extracellular L-Glu. Our data suggest the new hypothesis that activated microglia collude with astrocytes to cause the elevation of extracellular L-Glu in the early stages of neuroinflammation.

## Background

L-glutamate (L-Glu) is one of the most important excitatory neurotransmitters in the mammalian central nervous system (CNS). However, high concentrations of L-Glu cause excessive stimulation of L-Glu receptors and lead to neurotoxicity [1,2]. In astrocytes, GLAST (EAAT1 in humans) and GLT-1 (EAAT2) are the major functional L-Glu transporters in the CNS, and they play an important role in maintaining extracellular L-Glu concentrations below neurotoxic levels [3]. Impairments of L-Glu transporter function have been reported in numerous neurological diseases associated with inflammation, for example, Alzheimer’s disease [4], amyotrophic lateral sclerosis [5], major depressive disorder [6,7], and epilepsy [8-10]. Furthermore, elevated extracellular L-Glu content has been reported in *in vivo* and *in vitro* inflammation models [11,12]. Accordingly, the impairment of L-Glu transporters has been suggested to contribute to elevated extracellular L-Glu concentrations in inflammation; however, the specific role of such transporters remains unknown, as some inflammation models also cause cell death.

The CNS is composed of neurons and the following three types of glial cells: astrocytes, microglia, and oligodendrocytes [13]. Microglia are the primary cells that are activated in response to inflammatory stimulation [14,15] and are the resident innate immune cells in the CNS. Once activated, microglia exhibit a phenotypic switch from a resting ramified type to a motile amoeboid type [16,17] and release various soluble factors, including pro-inflammatory cytokines [18,19], reactive oxygen species [20], nitric oxide (NO) [16], L-Glu [21,22], and ATP [23,24]. Although the direct application of some of these factors has been reported to inhibit L-Glu transporters [25-28], few studies have examined the interaction between activated microglia and astrocyte L-Glu transporters in inflammation.

In this study, we aimed to clarify the interaction between activated microglia and astrocyte L-Glu transporters in inflammation. To quantify L-Glu transporter function, we measured the extracellular concentrations of L-Glu (that is, the concentration of L-Glu remaining) after a single exogenous application of L-Glu to the medium. To ensure that we measured the effects on live cells (and not L-Glu released from dying cells), we identified a condition of lipopolysaccharide (LPS) application that was suitable to induce inflammation without cell death. In this model, we found that activated microglia released L-Glu, the resultant elevation in extracellular L-Glu led to the elevation of intracellular L-Glu content in astrocytes through L-Glu transporters, and the increased level of intracellular L-Glu in astrocytes decreased GLAST expression. These reactions caused a further elevation of the extracellular concentration of L-Glu. Our data suggest a new hypothesis in which activated microglia collude with astrocytes to cause the elevation of extracellular L-Glu in the early stages of neuroinflammation.

## Methods

All procedures using live animals in this study were conducted in accordance with the guidelines of the National Institute of Health Sciences (NIHS), Japan, as developed under the Guide for the Care and Use of Laboratory Animals by the National Research Council. Also all experiments were approved by the ethics committee of the NIHS.

## Materials

L-Glu, LPS, CBX, anti-rabbit Iba-1 polyclonal antibody (019–19741), and paraformaldehyde (PFA) were purchased from Wako (Osaka, Japan). Dihydrokainic acid (DHK), adenosine 5′-triphosphate disodium salt hydrate (ATP), 2^′^ (3^′^)-O-(4-benzoylbenzoyl)ATP triethylammonium salt (BzATP), 2^′^,3^′^-O-(2,4,6-trinitrophenyl)ATP salt hydrate (TNP-ATP), adenosine 5^′^-triphosphate, periodate oxidized sodium salt (OxATP), poly-L-lysine hydrobromide, poly-ethylenimine, β-nicotinamide adenine dinucleotide (β-NAD), 3-(4,5-dimethyl-2-thiazolyl)-2,5-diphenyl-2H-tetrazolium bromide (MTT), 1-methoxy-5-methyl-phenazinium methyl sulfate (MPMS), Triton-X100, lactate lithium salt, anti-mouse β-actin monoclonal antibody (A5316), sodium deoxycholate, 2-mercaptoethanol, bromophenol blue sodium salt (BPB), and bovine serum albumin (BSA) were purchased from Sigma (St Louis, MO, USA). DL-threo-β-benzyloxyaspartic acid (TBOA) was purchased from TOCRIS (Ellisville, MO, USA). An MTT Cell proliferation assay kit was purchased from Life Technologies (Grand Island, NY, USA). Rat glutamate transporter (GLAST/EAAT1) control peptide (GLAST11-P) and rat glutamate transporter (GLT1/EAAT2) control peptide (GLT11-P) were purchased from Alpha Diagnostic (San Antonio, TX, USA). Clodronate disodium salt and polyoxyethylene (9) octylphenyl ether (NP-40) were purchased from Calbiochem (Darmstadt, Germany). Dulbecco’s modified eagle medium (DMEM), fetal bovine serum (FBS), and horse serum (HS) were purchased from GIBCO (Grand Island, NY, USA). Bovine liver glutamate dehydrogenase (GlDH) was purchased from Roche (Mannheim, Germany). RNeasy Mini Kits and an RNase-Free DNase set were purchased from Qiagen (Hilden, Germany). TaqMan one-step RT-PCR master mix reagents and TaqMan ribosomal RNA control reagents (VIC Prove) were purchased from Applied Biosystems (Foster City, CA, USA). 2-Amino-5,6,7,8-tetrahydro-4-(4-methoxyphenyl)-7-(naphthalen-1-yl)-5-oxo-4H-chromene-3-carbonitrile (UCPH-101), rabbit anti-GLAST polyclonal antibody (ab65978), and anti-chicken glial fibrillary acidic protein (GFAP) polyclonal antibody (ab4674) were purchased from Abcam (Cambridge, UK). Goat anti-EAAT2 (GLT1) antibody (sc-7760) was purchased from Santa Cruz Biotechnology (Santa Cruz, CA, USA). Anti-mouse Tuj1 (β3 tubulin) antibody (MAB5564) was purchased from Chemicon (Temecula, CA, USA). Donkey anti-rabbit IgG conjugated with horseradish peroxidase was purchased from Amersham Biosciences (Washington, DC, USA). The SuperSignal West Femto Trial Kit was purchased from Thermo Scientific (Rockford, IL, USA). The BCA protein assay kit was purchased from Pierce Chemical (Rockford, IL, USA). Tris (hydroxymethyl) aminomethane (Tris–HCl) was purchased from Bio-Rad (Hercules, CA, USA). Ethylenediaminetetraacetate (EDTA) and ethyleneglycoldiaminetetraacetate (EGTA) were purchased from Dojindo (Kumamoto, Japan). Goat serum, anti-mouse IgG-conjugated Alexa Fluor 488, anti-chicken IgG-conjugated Alexa Fluor 594, and anti-rabbit IgG-conjugated Alexa Fluor 647 were purchased from Vector Laboratories (Burlingame, CA, USA).

### Cell culture

#### Astrocyte-microglia-neuron mixed culture

The brains of 2-day-old Sprague–Dawley (SD) rats were aseptically removed, and the cerebral cortices were dissected. The tissues were dissociated by trituration and trypsinization. After centrifugation at 1,500 rpm for 5 min, the cells were suspended in DMEM supplemented with 10% FBS and 1% antibiotic-antimitotic agent, and the residual tissue aggregates were removed by filtration through a cell strainer with a pore size of 40 to 45 μm. The cells were seeded onto appropriately sized poly-L-lysine-coated plastic dishes or polyethyleneimine-coated cover glass, and grown for 8 days at 37°C in a humidified atmosphere containing 5% CO_2_. The medium was changed every 2 days.

#### Astrocyte culture

The primary culture of rat astrocytes was prepared according to a method previously described [29,30]. The cortical cells were obtained by the methods described above and were seeded in uncoated 75-cm^2 ^flasks at a density of 5 × 10^4 ^cells/cm^2^. The medium was changed 24 h after plating and then every 3 to 4 days. When the cells became confluent (10 to 14 days *in vitro* (DIV)), the non-astrocyte cells were detached from the flasks by shaking and removed by changing the medium. The remaining cells were dissociated by trypsinization and seeded onto appropriately sized poly-L-lysine-coated plastic dishes or polyethyleneimine-coated cover glass at a density of 3 × 10^5 ^cells/cm^2^. The cells became confluent again 2 to 3 days after plating.

#### Microglia culture

The primary culture of the rat microglia was prepared according to a method previously described [31]. In brief, the cells were obtained from the cerebral cortices of 1-day-old SD rats and seeded in poly-L-lysine-coated 75-cm^2^ flasks at a density of 2.5 × 10^5^ cells/cm^2^. The cells were then grown in DMEM containing 10% FBS, 10 U/mL penicillin, and 10 mg/mL streptomycin for 10 to 14 days at 37°C. The medium was changed every 2 to 3 days. The microglia were removed from the flask bottoms by gentle shaking (60 rpm, 2 min) and collected by centrifugation at 1,000 × *g* for 5 min. The microglia were resuspended and seeded at a density of 6 × 10^4 ^cells/cm^2 ^onto appropriately sized poly-L-lysine-coated plastic dishes or polyethyleneimine-coated cover glass.

#### Astrocyte-microglia co-culture

The microglia prepared by the methods described above were seeded at a density of 6 × 10^4 ^cells/cm^2 ^onto confluent astrocytes and cultured for 1 to 2 days.

#### Astrocyte-neuron co-culture

The astrocyte-microglia-neuron co-culture was treated with clodronate [32] at a concentration of 10 μg/mL for 4 days from 5 DIV to reduce the density of microglia to below 1.2 × 10^3 ^cells/cm^2^.

### Drug treatment

Stock solutions of 100 mM L-Glu, 10 μg/mL LPS, 100 μg/mL clodronate, 10 mM TBOA (non-selective L-Glu transporter inhibitor, IC50: 48 μM for GLAST, 7 μM for GLT-1), 10 mM UCPH-101 (a GLAST specific inhibitor, IC50: 0.66 μm for GLAST, >400-fold selectivity over EAAT2 and EAAT3), 10 mM DHK (a GLT-1-specific inhibitor, IC50: >3,000 μM for GLAST, 23 μM for GLT-1), 100 mM ATP, 10 mM BzATP, 10 mM, and 100 mM CBX in phosphate-buffered saline (PBS) were dissolved into the culture medium at the time of application. At 8 DIV, the astrocyte-microglia-neuron culture was treated with LPS at concentrations of 1 to 100 ng/mL for 6 to 72 h. TBOA, UCPH-101, or DHK was applied to the astrocyte-microglia-neuron co-culture for 24 h at 10 DIV. ATP or BzATP was applied to the astrocyte culture at concentrations of 100 to 3,000 μM or 10 to 300 μM, respectively, for 72 h. At 8 DIV, CBX was applied to the astrocyte-microglia-neuron culture at concentrations of 10 to 100 μM from 1 h before to the end of the LPS treatment.

### The measurement of the extracellular L-Glu concentration in the medium

The measurement of L-Glu concentration in the medium was performed according to a previously described method [33]. The culture medium in the 96-well plates was replaced with fresh medium containing 100 μM L-Glu. After 30 min, 50 μL of the culture medium in each well was collected. The L-Glu concentration was measured by mixing the medium with 50 μL of substrate mixture (20 U/mL GlDH, 2.5 mg/mL β-NAD, 0.25 mg/mL MTT, 100 μM MPMS, and 0.1% (vol/vol) Triton X-100 in 0.2 M Tris–HCl buffer (pH 8.2)) and incubating the mixture at 37°C for 30 min. The reaction was stopped by adding 100 μL of stop solution (50% (vol/vol) dimethylformamide and 20% (wt/vol) sodium dodecyl sulfate (SDS) in water (pH 4.7)). The amount of the reaction product (MTT formazan) was determined by measuring the absorbance at 570 nm (test wavelength) and 655 nm (reference wavelength) with a microplate reader. The extracellular L-Glu concentration was estimated from a standard curve, which was constructed for each assay using cell-free medium containing known concentrations of L-Glu. When the L-Glu was not applied after washing with fresh medium, no changes in extracellular L-Glu concentrations were observed in the 30 min incubation period in any experiments in this study. To measure the L-Glu released from microglia, the extracellular concentration of L-Glu was measured after 24 h of LPS treatment. The control values were almost same in the same culture batch but variable among different batches (40 to 60 μM). We therefore confirmed the reproducibility of the results in three independent experiments using different culture batches.

### LDH and MTT assays

The lactate dehydrogenase (LDH) activity in the medium was evaluated according to a previously described method [33]. Briefly, 50 μL of culture medium from each well of a 96-well plate was mixed with 50 μL of substrate mixture (2.5 mg/mL lactate lithium salt, 2.5 mg/mL β-NAD, 0.25 mg/mL MTT, 100 μM MPMS, and 0.1% (vol/vol) Triton X-100 in 0.2 M Tris–HCl buffer (pH 8.2)). After a 10 min incubation at 37°C, the reaction was stopped by adding 100 μL of stop solution as described above. The amount of MTT formazan was determined using a microplate reader. The data were normalized to the averaged value of the group treated with 0.1% (vol/vol) Triton-X 100 for 1 h. MTT reductions were evaluated according to the manufacturer’s instructions.

### Real-time quantitative polymerase chain reaction (TaqMan RT-PCR)

The total cellular ribonucleic acid (RNA) was extracted from cells with an RNeasy Mini Kit and treated with RNase-free DNase to eliminate genomic deoxyribonucleic acid (DNA) contamination. The amount of total RNA was quantified by measuring the OD260 using a Nanodrop spectrophotometer (Nanodrop, Wilmington, DE). The reactions (25 μL) contained 1 ng of total RNA, 900 nM forward and reverse primers, 250 nM TaqMan probe, and RNase inhibitor mix in the master mix solution. The RT-PCR was performed using the TaqMan One-Step RT-PCR master mix reagent kit according to the manufacturer’s protocol. The data were analyzed with 7,900 System SDS Software 2.2.2 (Applied Biosystems, Foster City, CA, USA) using the standard curve method. The GLAST and GLT-1 mRNA expression levels were normalized to the ribosomal RNA control (18S) expression levels. The primer sequences were as follows: 5’-GATCGGCATAATCATTGTCATCA-3’, 5’-CGATTTTACCTTCTCTGTACATGTTTC-3’ (GLAST), and 5’-CCGAGCTGGACACCATTGA-3’ 5’-AATGGACTGCGTCTTGGTCAT-3’ (GLT-1). Specific probes for GLAST (TCCACCCCGGAAAGGGCACG) and GLT-1 (CAACACCGAATGAATGCACGAAGACATCGA) were used.

### Western blotting

The cells were washed twice with PBS and once with lysate buffer (150 mM (wt/vol) NaCl, 10 mM (wt/vol) EDTA, 5 mM (wt/vol) EGTA, 0.5% mM (vol/vol) NP-40, and 0.5% (wt/vol) sodium deoxycholate in 10 mM Tris–HCl buffer (pH 7.4)). The protein concentration was measured using the BCA protein assay. The proteins (20 μg/lane) were mixed with SDS sample buffer (2% (wt/vol) SDS, 10% (vol/vol) glycerol, 0.25% (wt/vol) BPB, 5% (vol/vol) 2-mercaptoethanol in 125 mM Tris–HCl buffer (pH 6.8)), loaded onto a 10% polyacrylamide gel, electrophoresed, and transferred onto a PVDF membrane. The membrane was blocked with 5% (wt/vol) non-fat dry milk in Tris-buffered saline containing 0.1% (vol/vol) Tween 20. The membrane was incubated with rabbit anti-GLAST polyclonal antibody (1:4,000; ab65978, Abcam), mouse anti GLT-1 (1:2,000; sc-7760, Santa Cruz) or anti β-actin monoclonal antibody (1:5,000; A5316, Sigma-Aldrich) overnight at 4°C followed by incubation with the horseradish peroxidase-conjugated anti-rabbit antibody (1:20,000; Amersham Biosciences) or the anti-mouse or anti-goat antibody (1:20,000 Amersham Biosciences). The signals were scanned with an LAS3000 (Fuji Photo Film Co., Ltd., Tokyo, Japan) using an ECL western blot detection system (SuperSignal West Femto Trial Kit). For relative quantification of the expression levels of GLAST and GLT-1, we first compared the densities of the bands of the same amount of GLAST and GLT1 control proteins (full length) (1, 10 μg). In the LPS-exposure experiment, we normalized the band density of each subtype to the density of the 10 μg control band of the corresponding subtype. The bands of GLAST and GLT1 standard proteins were obtained at the same appropriate exposure time. The bands of GLAST and GLT1 in the LPS-exposure experiment were obtained at the same appropriate exposure time.

### Immunocytochemistry

The cells were washed with PBS three times and fixed with 4% PFA for 60 min at room temperature. After more washes with PBS, the cells were permeabilized and blocked for 60 min with 0.1% (vol/vol) Triton X-100, 5% (vol/vol) goat serum, and 1% (wt/vol) BSA in PBS. After washes with PBS, the cells were incubated with primary antibodies overnight at 4°C. Mouse monoclonal anti-Tuj1 antibody (1:500, MAB5564, Chemicon), chicken polyclonal anti-GFAP antibody (1:400, ab4674, Abcam), and rabbit polyclonal anti-Iba1 antibody (1:1,000, 019–19741, Wako) were used to stain neurons, astrocytes, and microglia, respectively. After washes with PBS, the cells were incubated with secondary antibodies (1:500, Invitrogen) conjugated to fluorochromes for 2 h at room temperature in the dark. After washes with PBS, fluorescent images of the cells were obtained by confocal microscopy (LSM5 Pascal, Zeiss).

### Characterization of microglial releasing factors that downregulate L-Glu transporters

For the conditioned medium study, the astrocyte-microglia-neuron mixed culture was treated with LPS (10 ng/mL, 72 h), and the conditioned medium was transferred to the astrocyte culture. After 72 h, the L-Glu clearance assay was performed on the astrocyte culture. In a separate experiment, the astrocyte culture was incubated for 72 h with a transwell carrying microglia that had been treated with LPS, and the L-Glu clearance assay was performed in the astrocyte culture. For the serial applications of L-Glu, the medium of the astrocyte-microglia-neuron mixed cultures was replaced with fresh medium containing L-Glu (100 μM) every 2 h for 24 h. The L-Glu clearance assay, TaqMan RT-PCR, and western blotting were performed after the serial application of L-Glu.

### The measurement of the astrocytic intracellular L-Glu concentration

The astrocyte-microglia co-culture was treated with LPS (10 ng/mL, 72 h) and washed twice with gentle shaking to remove microglia. After confirmation of the microglial removal under a microscope, 0.1% TritonX-100 was applied and incubated for 1 h. The L-Glu concentration in the supernatant was measured as described above. TBOA was applied from 1 h before the start of LPS treatment.

### Statistical analysis

All data are expressed as the mean ± the S.E.M. Statistical analyses were performed with Student’s *t* test or a one-way repeated-measures analysis of variance (ANOVA) followed by Tukey’s post-hoc test for multiple pairwise comparisons, as shown in the figure legends. In all of the comparisons, the differences were considered statistically significant when *P* <0.05. All of the experiments were repeated in triplicate, and the same results were obtained in all of the sessions.

## Results

### L-Glu uptake was decreased during inflammation without cell death through the downregulation of GLAST expression

**Figure 1:**
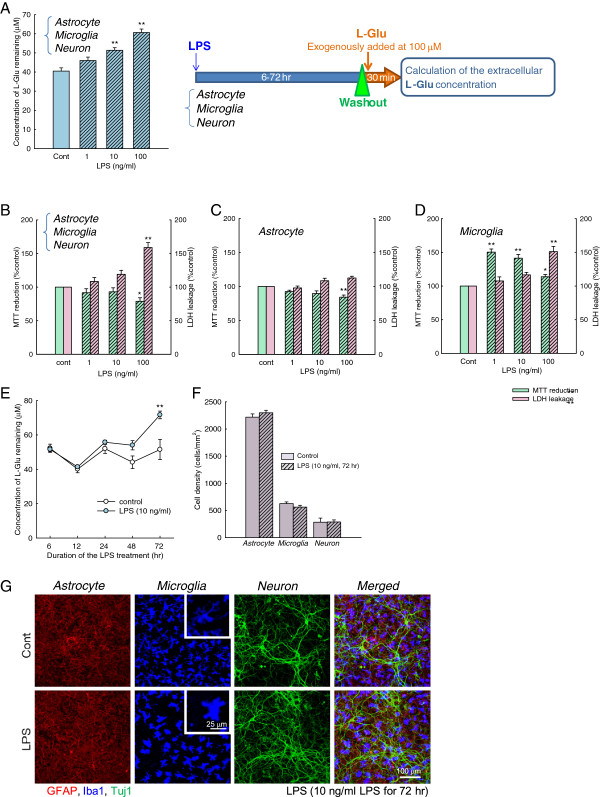
**The concentration of L-Glu remaining was increased during inflammation without cell death.** (**A**) Effects of LPS (1 to 100 ng/mL, 72 h) on the concentration of L-Glu remaining. The L-Glu remaining was significantly increased after incubation with 10 and 100 ng/mL LPS. **P < 0.01 *vs.* control group (*n* = 6), Tukey’s test following an ANOVA. (**B**, **C**, **D**) Effects of LPS on LDH leakage and MTT reduction in the astrocyte-microglia-neuron mixed cultures, astrocyte cultures, and microglia cultures. Significant LDH leakage and decreases in MTT reduction were induced by 100 ng/mL LPS but not by LPS concentrations <10 ng/mL in the mixed cultures (B). In the pure astrocyte culture, LPS concentrations <10 ng/mL affected neither LDH leakage nor MTT reduction (C). In the pure microglial culture, significant LDH leakage was induced by 100 ng/mL but not by LPS concentrations <10 ng/mL (**D**). (**E**) Time-dependent effects of LPS. The concentration of L-Glu remaining was measured after 6 to 72-h treatments with LPS (10 ng/mL). **P < 0.01 *vs.* control group (*n* = 6), paired *t*-test. (**F**) The effects of LPS (10 ng/mL, 72 h) on the number of neurons, astrocytes, and microglia. LPS treatment had no effect on the cell numbers (*n* = 5). (**G**) Immunostaining of astrocytes, microglia, and neurons in the mixed culture with antibodies against GFAP (red), Iba-1 (blue), and Tuj1 (green) after treatment with 10 ng/mL LPS for 72 h. LPS dramatically changed the shape of the microglia from a ramified shape to an amoeboid shape.

To definitively investigate the interactions between activated microglia and astrocytes, we used a mixed culture composed of astrocytes, microglia, and neurons. To quantify L-Glu transporter function, we measured the extracellular concentrations of L-Glu (that is, the concentration of L-Glu remaining) 30 min after a single exogenous application of L-Glu to the medium (the starting concentration was 100 μM). In this manner, we first determined the optimal conditions for inflammation without cell death. The cultures were treated with LPS for 72 h. The L-Glu remaining was significantly increased after incubation with 10 and 100 ng/mL LPS (Figure 1A). Significant LDH leakage and decreases in MTT reduction were induced by 100 ng/mL LPS but not by LPS concentrations less than 10 ng/mL (Figure 1B). In pure astrocyte cultures, significant decreases in MTT reduction were induced by 100 ng/mL LPS, but LPS concentrations less than 10 ng/mL did not affect either the LDH leakage or MTT reduction (Figure 1C). Whereas increases in MTT reduction were induced by 1 to 100 ng/mL LPS in pure microglial culture, significant LDH leakage was induced by 100 ng/mL but not by LPS concentrations <10 ng/mL (Figure 1D). When the treatment duration was changed, 10 ng/mL LPS was found to inhibit L-Glu uptake in a time-dependent manner, and a significant decrease was observed at 72 h (Figure 1E). Therefore, we adopted a 72-h treatment with 10 ng/mL LPS for inflammation without cell death. We also confirmed the morphology and cell density of each cell type in this inflammation model. LPS dramatically changed the shape of Iba-1 (+) microglia from a ramified shape to an amoeboid shape, which is consistent with the typical morphological changes observed after activation of microglia in previous reports [16,17] (Figure 1G). No changes were observed in astrocytes or neurons. The cell densities of all cell types did not change (Figure 1F).

**Figure 2:**
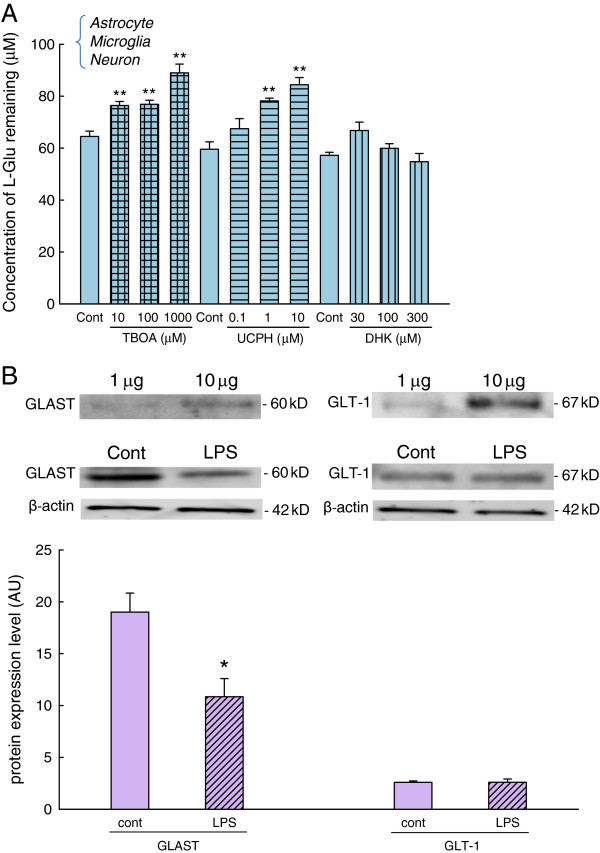
**The increase in the concentration of L-Glu remaining was mainly caused by the downregulation of GLAST.** (**A**) The effects of TBOA (30 to 300 μM), UCPH-101 (0.1 to 10 μM), and DHK (30 to 300 μM) on L-Glu clearance. Concentration-dependent inhibition was obtained by treatment with TBOA and UCPH-101. **P < 0.01 *vs.* the control group (*n* = 6), Tukey’s test following ANOVA. (**B**) The basal expression levels of astrocyte L-Glu transporters and the effect of LPS on their expression levels. Basally, the GLAST protein level is much higher than that of GLT1. The GLAST protein levels significantly decreased after the LPS treatment (10 ng/mL, 72 h), but GLT-1 protein levels did not change. *P < 0.05 *vs.* the control group (*n* = 4), Student’s *t*-test.

To determine which L-Glu transporter subtypes were responsible for the increase in the concentration of L-Glu remaining in the mixed cultures, we first examined the effects of various subtype-specific inhibitors on L-Glu uptake (Figure 2A). The cultures were treated with a non-selective L-Glu transporter inhibitor (TBOA) (10 to 1,000 μM), a GLAST-specific inhibitor (UCPH-101) (0.1 to 10 μM), or a GLT-1-specific inhibitor (DHK) (30 to 300 μM) for 24 h, and the concentration of L-Glu remaining in the medium 30 min after the application of L-Glu (the starting concentration was 100 μM) in the presence of the inhibitors was measured. TBOA and UCPH-101 increased the L-Glu remaining in a concentration-dependent manner to similar extents, whereas DHK did not. These results indicate that GLAST is the predominant functional transporter in the mixed culture. We therefore examined the basal expression levels of astrocyte L-Glu transporters and the effect of LPS on their expression levels. To compare the expression levels of GLAST and GLT1, we first compared the densities of western blotting bands for the same amount of GLAST and GLT1 control proteins (full length) (1, 10 μg) obtained at the same appropriate exposure time (Figure B, upper photos). Then, we quantified the expression levels of GLAST and GLT1 in the control- and LPS-treated mixed culture (Figure B, middle photos). The density of each band obtained at the same appropriate exposure time was normalized to the 10 μg control band of the corresponding subtype. Basally, the GLAST protein level is much higher than that of GLT1 (Figure 2, graph). The GLAST protein levels decreased to 65.7 ± 7.40% of control levels after LPS treatment (10 ng/mL, 72 h) (Figure 2C), but the GLT-1 protein level did not change. These results suggest that the LPS-induced increase in the L-Glu remaining was mainly caused by the downregulation of GLAST.

### Activated microglia caused the decrease in L-Glu uptake during inflammation without cell death

**Figure 3:**
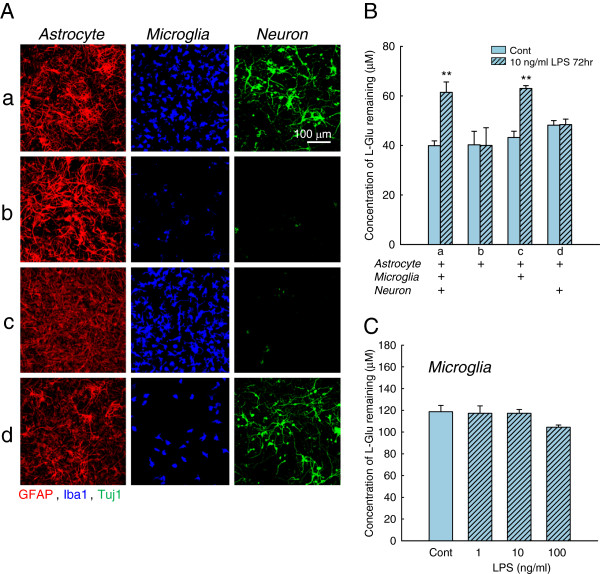
**Activated microglia are essential for the decrease in L-Glu uptake in the model of inflammation without cell death.** (**A**) Immunostaining of four types of cultures: astrocyte-microglia-neuron mixed culture (a), astrocyte culture (b), astrocyte-microglia co-culture (c), and neuron-astrocyte co-culture (d). (**B**) The effects of LPS (10 ng/mL, 72 h) on the concentration of L-Glu remaining in the four types of culture. Inhibitory effects were obtained in (a) and (c). **P < 0.01 *vs.* control group (*n* = 6), Student’s *t*-test. (**C**) The effects of LPS (10 ng/mL, 72 h) on the concentration of L-Glu remaining in microglial pure culture. The concentration of L-Glu remaining did not change during the assay (30 min).

To confirm that activated microglia are essential for the decrease in L-Glu uptake during inflammation without cell death, we examined the effects of LPS in four different types of cultures, including an astrocyte-microglia-neuron mixed culture (a), an astrocyte culture (b), an astrocyte-microglia co-culture (c), and an astrocyte-neuron co-culture (d) (Figure 3A). In (a), the astrocytes were confluent, and the cell densities of the microglia and the neurons were 3.0 × 10^4 ^cells/cm^2 ^and 6.0 × 10^4 ^cells/cm^2^, respectively. Therefore, the cell density of the microglia in (c) and that of neurons in (d) were carefully adjusted to 3.0 × 10^4^ cells/cm^2^ and 6.0 × 10^4 ^cells/cm^2^, respectively. Furthermore, in (b) to (d), we confirmed that the density of each cell type that had been presumably removed was sufficiently low; the number of microglia in (b) and (d) was <1.2 × 10^3 ^cells/cm^2^, and the number of neurons in (b) and (c) was <1.0 × 10^3 ^cells/cm^2^. Astrocytes were confluent in (a) to (d). When we treated these cultures with LPS (10 ng/mL, 72 h), significant decreases in L-Glu uptake occurred in (a) and (c) but not in (b) nor (d). As shown in Figure 3B (b), the L-Glu uptake in astrocyte pure culture was not changed by LPS. We further confirmed that in the LPS-treated microglial pure culture, the concentration of L-Glu remaining did not change during the assay (30 min) (Figure 3C). These results indicate that the increase in L-Glu remaining, that is, the inhibition of L-Glu uptake, observed in Figure 3B (a) and (c) was caused by the interaction between the activated microglia and the astrocytes during 72 h of LPS treatment.

### L-Glu released from activated microglia caused the downregulation of GLAST expression during inflammation without cell death

**Figure 4:**
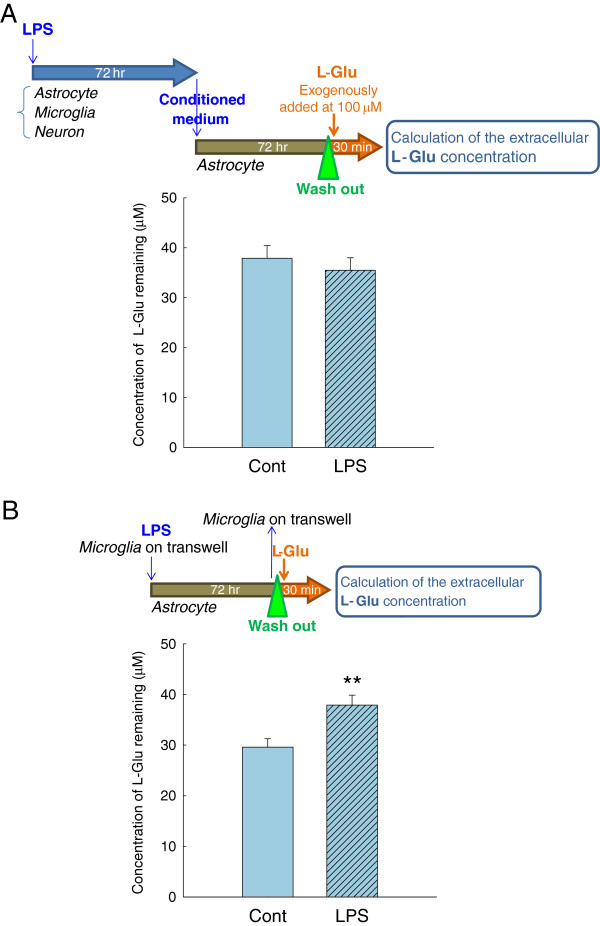
**Transwell carrying activated microglia, but not the conditioned medium, decreased L-Glu uptake in the astrocyte culture.** (**A**) Effect of the conditioned medium collected from the astrocyte-microglia-neuron mixed culture treated with LPS (10 ng/mL, 72 h) on L-Glu clearance in the astrocyte culture. A 72-h incubation with the conditioned medium did not affect L-Glu clearance in the astrocyte culture. (**B**) The effects of transwell-cultured microglia in the presence of LPS (10 ng/mL, 72 h) on L-Glu uptake in the astrocyte culture. A significant decrease in the L-Glu uptake was caused by 72-h treatment with the transwell. **P < 0.01 *vs*. control group (*n* = 6), Student’s *t*-test.

**Figure 5:**
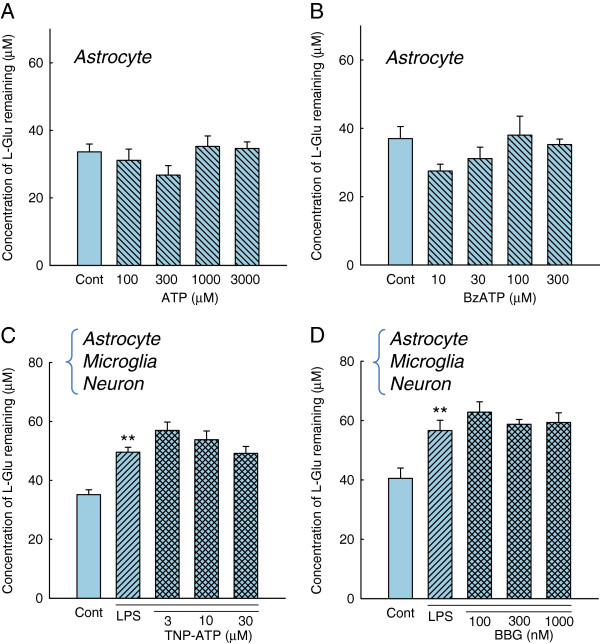
**ATP did not contribute to the downregulation of GLAST in the inflammation without cell death.** (**A**, **B**) Neither ATP nor the P2X7 agonist BzATP affected L-Glu uptake. (**C**, **D**) Neither the P2X receptor antagonist TNP-ATP nor the P2X7 specific antagonist BBG affected the LPS-induced decrease in L-Glu uptake. **P < 0.01 *vs.* control group (*n* = 6), Tukey’s test following ANOVA.

**Figure 6:**
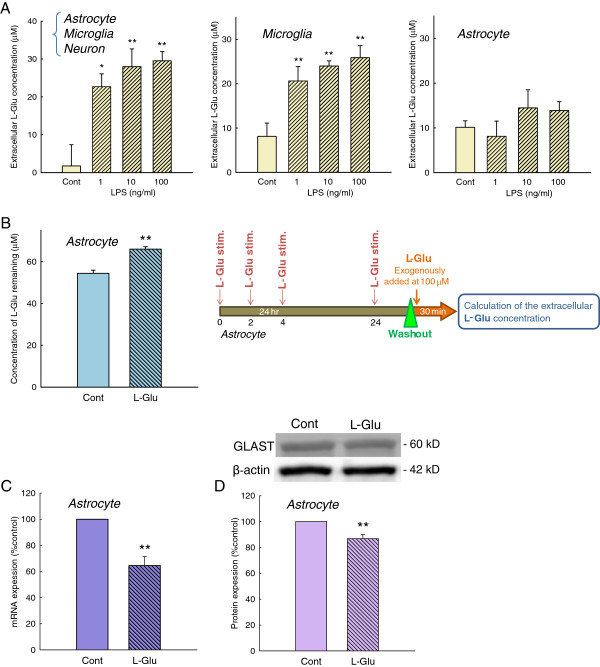
**L-Glu released from activated microglia caused the downregulation of GLAST.** (**A**) Effects of LPS (1 to 100 ng/mL, 24 h) on the extracellular concentration of L-Glu in the astrocyte-microglia-neuron mixed culture (left), microglia culture (center), and astrocyte culture (right). LPS significantly increased the extracellular L-Glu in the astrocyte-microglia-neuron mixed culture and the microglia culture. *P < 0.05, **P < 0.01 *vs.* control group (*n* = 6), Tukey’s test following ANOVA. (**B**) The effect of sustained exposure to L-Glu on L-Glu clearance in the astrocyte culture. The culture medium was changed to fresh medium containing 100 μM L-Glu every 2 h. L-Glu clearance was suppressed by sustained exposure to L-Glu for 24 h. **P < 0.01 *vs*. the control group (*n* = 6), Student’s *t*-test. (**C**) The effect of sustained exposure to L-Glu on GLAST mRNA levels. Sustained exposure to L-Glu decreased GLAST mRNA. **P < 0.01 *vs.* control group (*n* = 4), Student’s *t*-test. (**D**) The effect of sustained exposure to L-Glu on GLAST protein levels. Sustained exposure to L-Glu decreased the protein expression of GLAST. **P < 0.01 *vs.* control group (*n* = 5), Student’s *t*-test.

**Figure 7:**
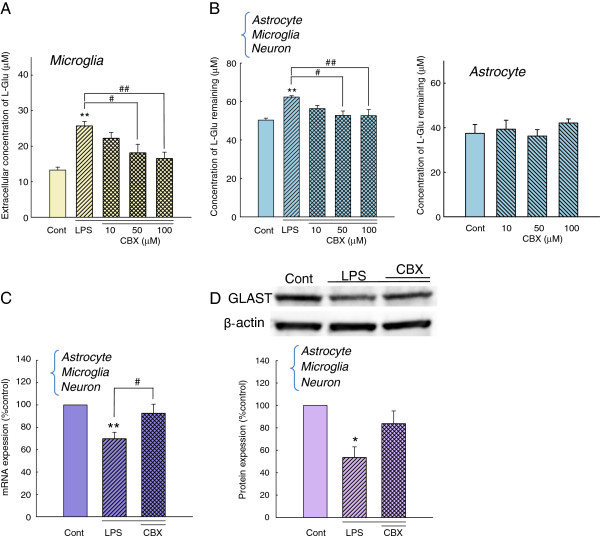
**CBX ameliorated the decrease in L-Glu uptake and GLAST expression in inflammation without cell death.** (**A**) The effect of CBX on L-Glu release from activated microglia. CBX concentration-dependently suppressed L-Glu release due to LPS (10 ng/mL, 24 h)-activated microglia. **P < 0.01 *vs*. control group, #*P* < 0.05, ##*P* < 0.01 *vs*. the LPS-treated group (*n* = 5), Tukey’s test following ANOVA. (**B**) The effects of CBX on the decreased L-Glu uptake in the model of inflammation without cell death. CBX concentration-dependently inhibited the LPS (10 ng/mL, 72 h)-induced decrease in L-Glu uptake in the astrocyte-microglia-neuron mixed culture (left) but not in the astrocyte culture (right). **P < 0.01 *vs*. control group, #*P* < 0.05, ##*P* <0.01 *vs*. the LPS-treated group (*n* = 5), Tukey’s test following ANOVA. (**C**) The effect of CBX on the LPS-induced decrease in GLAST mRNA levels. In the presence of CBX (100 μM), the LPS (10 ng/mL, 24 h)-induced decrease in GLAST mRNA level was ameliorated in the astrocyte-microglia-neuron mixed culture. **P < 0.01 *vs*. control group, #*P* < 0.05 *vs*. the LPS-treated group (*n* = 5), Tukey’s test following ANOVA. (**D**) The effect of CBX on the LPS-induced decrease in GLAST protein levels. In the presence of CBX (100 μM), the LPS (10 ng/mL, 72 h)-induced decrease in GLAST protein levels was ameliorated in the astrocyte-microglia-neuron mixed culture. *P < 0.05 *vs*. control group (*n* = 4), Tukey’s test following ANOVA.

Activated microglia release various soluble factors in inflammatory processes [16,18-24]. To examine the involvement of these factors in the action of activated microglia on L-Glu transporters, we applied the conditioned medium collected from the inflammation without cell death model (Figure 4A) to a culture of astrocytes alone. A 72 h-incubation with the conditioned medium did not affect the L-Glu uptake in the astrocyte culture. We then incubated the astrocyte culture with a transwell on which microglia were cultured in the presence of LPS (10 ng/mL, 72 h) (Figure 4B). Notably, a significant decrease in L-Glu uptake was obtained under these conditions. Because LPS in this condition did not directly affect the L-Glu uptake in the astrocyte culture, as shown in Figure 3B (b), these results suggest that the secreted factors are released from microglia and are degraded or taken up after their release. ATP has been shown to downregulate GLAST through the P2X7 receptor [28] and the ectonucleotidases of astrocytes rapidly convert extracellular ATP to ADP, ultimately to AMP [34]. We first examined the contribution of ATP to the downregulation of GLAST in the inflammation model without cell death. Exogenous application of ATP (Figure 5A) and P2X7 agonist BzATP (Figure 5B) did not change the L-Glu uptake. We also confirmed that neither the P2X receptor antagonist TNP-ATP (Figure 5C) nor the P2X7-specific antagonist BBG (Figure 5D) inhibited the decrease in L-Glu uptake in this inflammation model. We then examined the possibility of L-Glu. L-Glu is released by activated microglia through hemichannels [21,22] and taken up by L-Glu transporters after its release. We hypothesized that the secreted factor may be L-Glu. We first examined whether L-Glu was indeed released from microglia during inflammation without cell death. As shown in Figure 6A, left, LPS elevated the extracellular L-Glu concentrations in the astrocyte-microglia-neuron mixed cultures, and a significant elevation was observed even at a concentration of 1 ng/mL (Figure 6A left). An elevation of the extracellular L-Glu concentration was observed in the microglia culture (Figure 6A center) but not in the astrocyte culture (Figure 6A right). These results indicate that L-Glu was released from activated microglia during inflammation without cell death. To confirm our hypothesis, we tested the effect of the sustained elevation of extracellular L-Glu on L-Glu uptake in the mixed culture. To yield a sustained elevation of extracellular L-Glu, the culture medium of the astrocyte culture was freshly supplemented with 100 μM L-Glu every 2 h for 24 h, as preliminary studies showed that a concentration of 100 μM extracellular L-Glu was reduced to almost zero after 4 h in confluent astrocyte cultures (not shown). As shown in Figure 6B, the sustained elevation of extracellular L-Glu resulted in a significant decrease in L-Glu uptake in the astrocyte culture. GLAST expression was significantly decreased at the mRNA level (Figure 6C) and the protein level (Figure 6D) by the same treatment. These results suggest that L-Glu was responsible for the decrease in L-Glu uptake during inflammation without cell death. When the microglia cultures were treated with LPS (10 ng/mL, 24 h) in the absence or presence of the hemichannel inhibitor, CBX (10 to 100 μM), the L-Glu release from the activated microglia was suppressed in a concentration-dependent manner (Figure 7A). CBX (100 μM) almost completely prevented the LPS-induced (10 ng/mL, 72 h) decrease in L-Glu uptake in the mixed culture (Figure 7B, left) but had no effect in the astrocyte culture (Figure 7B, right). Furthermore, CBX reversed the LPS-induced down-regulation of GLAST expression at the mRNA (Figure 7C) and protein levels (Figure 7D).

**Figure 8:**
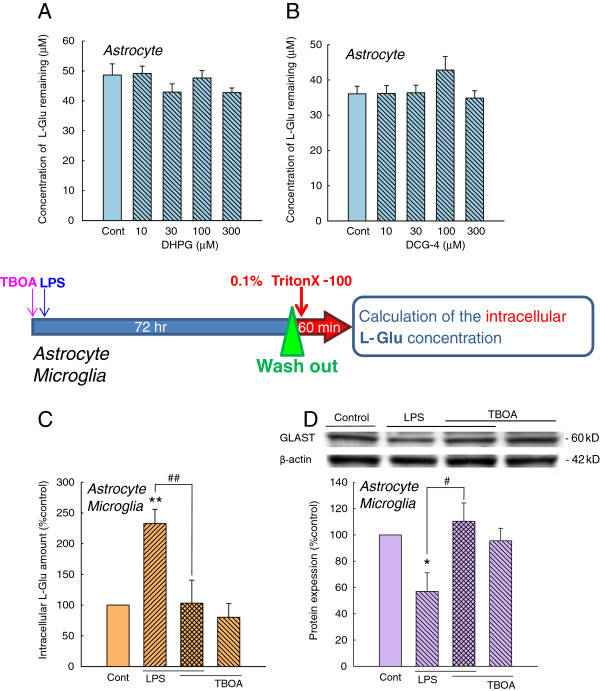
**The elevation of intracellular L-Glu in astrocytes is important for the downregulation of GLAST.** (**A**, **B**) The effects of mGluR agonists on L-Glu uptake. Neither the group I mGluR agonist DHPG (A) nor the group II mGluR agonist DCG-4 (B) affected L-Glu uptake. (**C**) The amount of astrocytic intracellular L-Glu after LPS-treatment in the absence or presence of TBOA in astrocyte-microglia co-cultures. LPS significantly increased the amount of astrocytic intracellular L-Glu, and TBOA completely suppressed this increase. **P < 0.01 *vs*. control group, ##*P* < 0.01 *vs.* the LPS-treated group (*n* = 6), Tukey’s test following ANOVA. (**D**) The astrocytic expression of GLAST after LPS treatment in the absence or presence of TBOA in astrocyte-microglia co-cultures. TBOA suppressed the downregulation GLAST caused by LPS. *P < 0.05 *vs.* control group, #*P* < 0.05 *vs*. the LPS-treated group (*n* = 6), Tukey’s test following ANOVA

We next tried to clarify the mechanisms through which the sustained elevation of extracellular L-Glu downregulates GLAST. Recent reports have suggested that the expression of L-Glu transporters is regulated by L-Glu through metabotropic glutamate receptors (mGluRs). We therefore first examined the involvement of metabotropic glutamate receptors (mGluRs). Neither the group I mGluR agonist DHPG nor the group II mGluR agonist DCG-4 affected either L-Glu uptake (Figure 8A and B) or the expression level of GLAST (not shown). Sustained elevation of extracellular L-Glu caused by activated microglia is expected to cause the elevation of intracellular L-Glu in astrocytes. We therefore examined whether the elevation of intracellular L-Glu itself is important for the downregulation of GLAST. To do this, we first measured the amount of astrocytic intracellular L-Glu after LPS-treatment in the absence or presence of TBOA in astrocyte-microglia co-cultures (Figure 8C). LPS significantly increased the amount of intracellular L-Glu, and TBOA completely suppressed this increase. Western blotting showed that TBOA suppressed the downregulation GLAST caused by LPS (Figure 8D). TBOA itself did not have effects on either the amount of intracellular L-Glu or the GLAST protein level. These results indicate that the elevation of astrocytic intracellular L-Glu, but not the signaling cascade from the cell surface, is important for the downregulation of GLAST.

**Figure 9:**
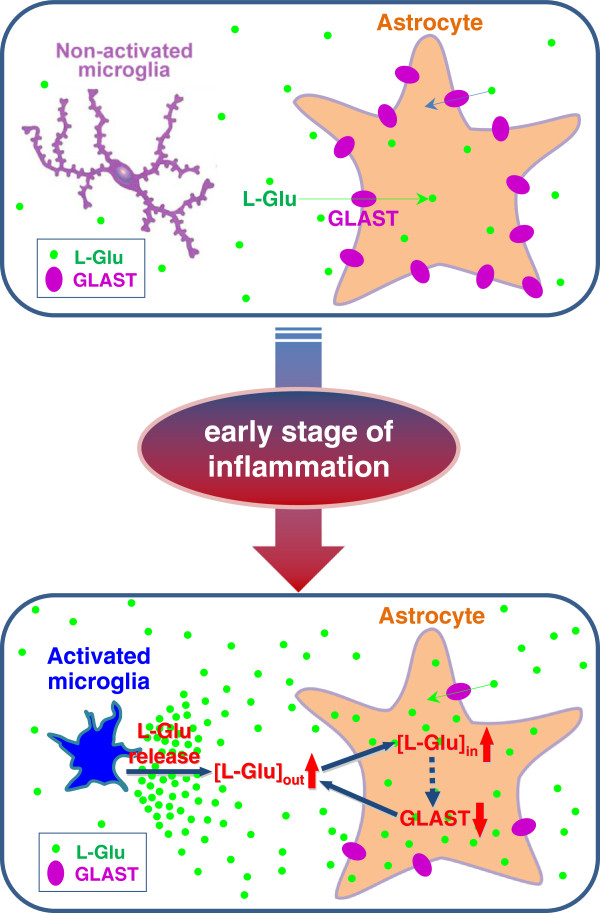
**Schematic model of the ‘collusion’ hypothesis for the elevation of extracellular L-Glu in the early stages of inflammation.**1. Activated microglia release L-Glu. 2. The resultant elevation of extracellular L-Glu causes the elevation of astrocytic intracellular L-Glu. 3. The elevation of astrocytic intracellular L-Glu downregulates GLAST expression. 4. The decrease in GLAST expression further exacerbates the elevation of extracellular L-Glu

Our findings suggest that activated microglia trigger the elevation of extracellular L-Glu through their own release of L-Glu, astrocyte L-Glu transporters are downregulated by the elevation of astrocytic intracellular L-Glu, and further elevation of extracellular L-Glu occurs early in neuroinflammation. A schematic model of this ‘collusion’ hypothesis is shown in Figure 9.

## Discussion

To quantify L-Glu transporter function, we measured the extracellular concentrations of L-Glu 30 min after a single exogenous application of L-Glu to the medium (the starting concentration was 100 μM). To limit any contributions of extra L-Glu from dying cells, and to verify a substantial contribution of the decrease in L-Glu transport potency to an elevated concentration of extracellular L-Glu in inflammation, we first determined the optimal conditions for inflammation without cell death. We used a lower concentration of LPS (10 ng/mL) than is generally used [35,36]. LPS application at a concentration of 10 ng/mL for 72 h activated the microglia but did not cause either LDH leakage or decreases in MTT reduction in the mixed culture, astrocyte pure culture, or microglia pure culture. LPS induces an inflammatory response in microglia via Toll-like receptor 4 (TLR4) [37]. TLR4 is also expressed by astrocytes, and astrocytes themselves have shown inflammatory responses in response to LPS in some reports [38]. In the present study, however, microglia were essential for the decreased L-Glu by astrocytes, and LPS did not affect L-Glu uptake in astrocyte cultures. Because the expression of TLR4 by astrocytes is less than that of microglia [37], the LPS stimulation in our model of inflammation without cell death may be insufficient to induce phenotypic changes in astrocytes. These mild inflammatory conditions may reflect the early stages of neuroinflammation *in vivo*, in which early microglial activation has been observed to precede the phenotypic changes in astrocytes [39].

In the present study, we pharmacologically confirmed that GLAST, and not GLT-1, was the predominant functional L-Glu transporter. We also confirmed that the expression level of GLT-1 is much lower than that of GLAST. GLT-1 has been reported to be functional in neuron-astrocyte co-cultures at 32 to 44 DIV [40]. This discrepancy most likely arises from the maturation stages of neurons, as the functional development of GLT-1 correlates with neuronal maturation [41]. The expression of GLAST was significantly decreased in the ‘non-cell death inflammation model’, which indicates that the decrease in L-Glu uptake in this inflammation model was mainly caused by the downregulation of GLAST.

Activated microglia release various soluble factors, including inflammatory cytokines [18,19], reactive oxygen species [20], NO [16], L-Glu [21,22], and ATP [23,24]. We demonstrated that L-Glu is the factor that downregulates GLAST in astrocytes during inflammation without cell death. Although activated microglia are known to release L-Glu through hemichannels [21,22], the neurological importance of this phenomenon remains unclear. We showed that the hemichannel inhibitor CBX completely suppressed the release of L-Glu from microglia, the decrease in L-Glu uptake, and the down-regulation of GLAST expression during inflammation without cell death. These data provide strong evidence that L-Glu is the microglial releasing factor that downregulates GLAST. High concentrations of ATP have also been shown to downregulate GLAST through the P2X7 receptor [28]. However, we believe that ATP did not contribute to the down-regulation of GLAST in the inflammation model without cell death here because L-Glu uptake did not change when the astrocyte culture was treated with ATP (Figure 5A) or the P2X7 agonist BzATP (Figure 5B). We also confirmed that neither the P2X receptor antagonist TNP-ATP (Figure 5C) nor the P2X7-specific antagonist BBG (Figure 5D) inhibited the decrease in L-Glu uptake in this inflammation model. Other microglial releasing factors, such as TNF-α, IL-1β, and arachidonic acid, are also known to decrease the L-Glu transport in astrocyte cultures [25-27]. However, the conditioned media collected from our model of inflammation without cell death had no effect in the astrocyte culture. Because the LPS stimulation here was lower than that of other studies [35,36] (to prevent cell death), the amount of these factors in the conditioned media may have been insufficient to affect L-Glu transporters.

Recent reports have suggested that the expression of L-Glu transporters is regulated by L-Glu through metabotropic glutamate receptors (mGluRs), that is, the group I mGluR agonist downregulates GLAST, whereas the group II mGluR agonist has the opposite effect [42,43]. However, neither the group I mGluR agonist nor the group II mGluR agonist affected the expression of GLAST in the present study. Instead, we clarified that the elevation of intracellular L-Glu in astrocytes is important for the downregulation of GLAST as shown in Figure 8. It has been clarified that translation initiation is regulated by intracellular L-Glu transported by GLAST in Bergmann glial cells [44,45]. They also showed that mammalian target of rapamycin (mTOR), increase in intracellular Ca^2+ ^levels, and p60(Src)/PI3K/PKB pathway are involved in this regulation. Further investigation is necessary to confirm whether the same pathways are involved in the downregulation of GLAST observed in our study. Of interest, a sustained elevation of extracellular L-Glu induced by the same protocol as Figure 6 did not cause the downregulation of glutamine synthetase (GS) in our preliminary experiment (data not shown), suggesting that this regulation is GLAST or L-Glu transporter-specific. The comparison of the upstream DNA sequences of GLAST and GS might provide useful information. Besides, in *Saccharomyces cerevisiae*, the activator (NIL1p) of the amino acid transporter is inactivated by increases in intracellular glutamate [46]. It is possible that a conserved mechanism similar to this also exist in astrocytes. Our findings strongly suggest that L-Glu is the microglial releasing factor which results in downregulation of GLAST in the early stage of inflammation. However, whether or not the quantity of L-Glu released from microglia is enough to induce a range of reaction still needs to be elucidated. Based on the discussion above, the co-factors to enhance the signaling pathway in the astrocytes leading to the downregulation of GLAST might be also released from microglia.

## Conclusions

Our findings suggest that activated microglia trigger the elevation of extracellular L-Glu through their own release of L-Glu, astrocyte L-Glu transporters are downregulated by the elevation of astrocytic intracellular L-Glu, and further elevation of extracellular L-Glu is caused as an early event of neuroinflammation (Figure 9).

## Abbreviations

ANOVA: Analysis of variance; ATP: Adenosine 5^′^-triphosphate disodium salt hydrate; BPB: Bromophenol blue sodium salt; BSA: Albumin bovine serum; BzATP: 2^′ ^(3^′^)-O-(4-Benzoylbenzoyl)ATP triethylammonium salt; CBX: Carbenoxolone; CNS: Central nervous system; DHK: Dihydrokainic acid; DIV: Days *in vitro*; DMEM: Dulbecco’s modified eagle medium; DNA: Deoxyribonucleic acid; EDTA: Ethylenediaminetetraacetate; EGTA: Ethyleneglycoldiaminetetraacetate; FBS: Fetal bovine serum; GFAP: Glial fibrillary acidic protein; GlDH: Glutamate dehydrogenase; HS: Horse serum; LDH: Lactate dehydrogenase; L-Glu: L-glutamate; LPS: Lipopolysaccharide; mGluRs: Metabotropic glutamate receptors; MPMS: 1-methoxy-5-methyl-phenazinium methyl sulfate; MTT: 3-(4,5-dimethyl-2-thiazolyl)-2,5-diphenyl-2H-tetrazolium bromide; β-NAD: β-nicotinamide adenine dinucleotide; NO: Nitric oxide; NP-40: Polyoxyethylene(9)octylphenyl ether; OxATP: Adenosine 5^′^-triphosphate periodate oxidized sodium salt; PBS: Phosphate-buffered saline; PFA: Paraformaldehyde; RNA: Ribonucleic acid; RT-PCR: Polymerase chain reaction; SD: Sprague–Dawley; SDS: Sodium dodecyl sulfate; TBOA: DL-threo-β-benzyloxyaspartic acid; TLR4: Toll-like receptor 4; TNP-ATP: 2^′^,3^′^-O-(2,4,6-Trinitrophenyl)ATP salt hydrate; Tris–HCl: Tris (hydroxymethyl) aminomethane; Tuj1: β3 tubulin; UCPH 101: 2-Amino-5,6,7,8-tetrahydro-4-(4-methoxyphenyl)-7-(naphthalen-1-yl)-5-oxo-4H-chromene-3-carbonitrile.

## Competing interests

I declare that I have no significant competing financial, professional or personal interests that might have influenced the performance or presentation of the work described in this manuscript.

## Authors’ contributions

JT performed experimental work and manuscript writing. KF performed experimental work. MM performed additional experimental work. TS provided advice on the experimental direction. YS provided advice on manuscript writing and preparation. KS designed the biological experimental plan and performed biological experiments, data analysis, manuscript writing, and preparation. All authors have read and approved the final version of the manuscript.
